# Dynamic colour change in zebrafish (*Danio rerio*) across multiple contexts

**DOI:** 10.1098/rsos.241073

**Published:** 2025-01-08

**Authors:** Ella J. Ackroyd, Robert J. P. Heathcote, Christos C. Ioannou

**Affiliations:** ^1^ School of Biological Sciences, University of Bristol, Bristol, UK; ^2^ School of Biological and Marine Sciences, University of Plymouth, Plymouth, UK; ^3^ Department of Biology, University of Oxford, Oxford, UK

**Keywords:** dynamic colour change, background matching, signalling, dazzle colouration, zebrafish

## Abstract

Many animals are capable of rapid dynamic colour change, which is particularly well represented in fishes. The proximate mechanisms of dynamic colour change in fishes are well understood; however, less attention has been given to understanding its ecological relevance. In this study, we investigate dynamic colour change in zebrafish (*Danio rerio*) across multiple contexts, using a protocol to image the colouration of live fish without anaesthesia under standardized conditions. We show that zebrafish respond to different visual environments by darkening their overall colouration in a dark environment and lightening in a light environment. This is consistent with crypsis through background matching as a function of dynamic colour change. Additionally, we find that zebrafish use dynamic colour change to increase the internal contrast of their striped patterning in the presence of conspecifics. We speculate that this may function in social signalling and/or dazzle colouration. We find no effect of a predator stimulus on dynamic colour change. Finally, we discuss the potential for zebrafish to use multiple colouration strategies simultaneously as distance-dependent effects, considering the typical viewing distances of zebrafish and their predators.

## 1. Introduction

An incredible phenotypic diversity of colour is observed across the 32 000 or so described species of fish. Fish colouration is determined by the arrangement of chromatophore cells throughout the dermal integument. Chromatophore cells contain either pigment granules that absorb certain wavelengths of visible light (e.g. melanophores and xanthophores), or crystalline structures that reflect certain wavelengths (e.g. iridophores) [1,2]. Many species of fish are capable of dynamic colour change, which represents changes in colouration that are reversible and often rapid. Dynamic colour change can occur through two mechanistic pathways, relating either to changes in overall chromatophore abundance, or to changes in pigment and crystalline structure distribution within the chromatophores. These processes are termed morphological and physiological colour change, respectively. Physiological colour change in fishes can be neurally or hormonally regulated [3–5], but is differentiated from morphological colour change (which is exclusively hormonally regulated) by the speed of the chromatophore response; physiological colour change occurs rapidly over seconds to minutes, whilst morphological colour change occurs over days to months [6]. The mechanisms driving morphological colour change in fishes are well documented, and our mechanistic understanding of physiological colour change in fishes has benefited from recent attention [5,7,8]. What is less well understood, however, are the ecological functions of dynamic colour change in fishes [4].

Here, we investigate the potential ecological functions of dynamic colour change in zebrafish (*Danio rerio*). Owing to their historic and ongoing popularity as a developmental and genetic model species, the mechanisms underlying the development of zebrafish colouration and pattering are well understood [9–11]. The mechanisms of dynamic colour change have also been previously investigated in zebrafish [4]; morphological colour change in zebrafish is known to occur through the regulation of melanophore density by melanin-concentrating hormone (MCH) and melanocyte-stimulating hormones (MSH), inducing long-term colour change [6,12]. Physiological colour change occurs in zebrafish through the intracellular motility of melanin pigment granules within melanophores [4], where dispersal of the pigment causes darker colouration, and aggregation of the pigment causes lighter colouration. Zebrafish are a relevant model in which to explore the adaptive functions of dynamic colour change, given that despite our current understanding of the mechanisms driving dynamic colour change in zebrafish, its ecological relevance is unclear.

Several previous investigations of dynamic colour change in fishes suggest that a likely ecological function is to facilitate crypsis against multiple backgrounds [13–16]. This frequently takes the form of background matching: a widespread cryptic colouration strategy in which an animal’s colouration closely reflects that of their visual environment [17]. Across their native South Asian range, wild zebrafish are found across numerous habitat and microhabitat types; zebrafish typically occupy standing water bodies such as ponds, rice paddies and drainage ditches and also riverine habitats with fast-flowing water [18,19], where they are evenly distributed throughout the water column, and frequently occupy a full spectrum of vegetation cover [20]. Variation in ecological factors (such as water depth, water quality and vegetation cover) between habitats and microhabitats lends to visual heterogeneity in the zebrafish’s natural environment, and so zebrafish predators are likely to view these fish against multiple backgrounds. By changing colour to more closely resemble these multiple backgrounds, zebrafish may be able to achieve crypsis across their entire environment, providing an adaptive advantage. Previous work has demonstrated that zebrafish are capable of both long- and short-term ‘background adaptation’ to their visual environment via dynamic colour change, where individuals lighten or darken their colouration to more closely match a light or dark visual background [4,6,21]. Despite some suggestions that such background adaptation may function in crypsis [4], the response has exclusively been investigated in relation to the proximate mechanisms underlying the colour change, rather than from a behavioural perspective. Here, we suggest that crypsis through background matching is a probable ecological function of dynamic colour change in zebrafish. We investigate this by characterizing zebrafish colouration following exposure to a light or dark visual environment, predicting that dynamic colour change will facilitate background matching through directional changes in zebrafish luminance, with fish becoming darker in the dark environment and lighter in the light environment. In some species, cryptic colouration as mediated by dynamic colour change appears to be facultative. For example, dynamic colour change for crypsis in the dwarf chameleon (*Bradypodion* spp.) occurs more often in the presence of predators than in the absence of predators [22], with several dwarf chameleon species using different camouflage strategies in response to different predatory threats [23,24]. As such, we also compare the dynamic colour change in zebrafish in the presence and absence of a predatory stimulus across both light and dark visual environments. If zebrafish perform facultative crypsis, we expect that the fish will show an enhanced colour change response when presented with predatory stimuli compared with when a predator is absent, in order to maintain facultative crypsis across different visual environments.

In addition to crypsis, dynamic colour change functions in transient social signalling in several taxa [7]. Social signalling is important in facilitating the social recognition of conspecifics, which, in turn, is essential for collective behaviour [25,26]. Shoaling is a form of collective behaviour that allows fish to form and maintain cohesive groups, and facilitates foraging and predator avoidance [27]. As shoaling is typically mediated by vision in diurnal freshwater fish [28], colouration and patterning are likely to play an important role in social signalling as associated with shoaling behaviour. Horizontal stripes in particular are strongly associated with sociality in fishes and may function as social signals for many fish species [29]. In the western rainbowfish (*Melanotaenia australis*), there is some evidence that dynamic colour change interacts with assortative shoaling preferences; individuals with a darker or lighter phenotype, as determined by dynamic colour change, prefer to shoal with other darker or lighter individuals, respectively [30]. Assortative shoaling by colour pattern is thought to counter the oddity effect, thus reducing predation risk for shoaling individuals [31,32]. Zebrafish stripes are known to be an important shoaling cue, where fish prefer to shoal assortatively with similarly patterned individuals [33,34]. However, it is not yet understood if dynamic colour change might play a role in mediating these cues. Dynamic colour change also frequently functions in social signalling to mediate agonistic interactions, for instance by signalling social subordination, as in the Arctic charr (*Salvelinus alpinus*) [35], or motivation and fighting ability, as in the veiled chameleon (*Chamaeleo calyptratus*) [36]. There is some speculation that zebrafish stripes may signal social information during both agonistic and shoaling interactions [37]. We therefore suggest that social signalling is another likely ecological function of dynamic colour change in zebrafish. Here, we investigate this by measuring zebrafish colouration in response to a social stimulus across both the light and dark visual environments. We predict that any changes in zebrafish colouration as related to social signalling will manifest as changes in the internal contrast between the fish’s light and dark stripes, owing to the potential use of high-contrast striped patterns as social signals in several species including zebrafish [29,37].

Conspicuous signalling and crypsis represent two potential functions of dynamic colour change that are often in direct conflict with one another [38]. Here, we directly compare zebrafish colouration in the presence or absence of predators or conspecifics for an insight into how zebrafish might navigate such a conflict.

## 2. Methods

### 2.1. Subjects and husbandry

Ninety-nine wild-type adult zebrafish (*D. rerio*), sourced from a UK-based ornamental fish supplier (Maidenhead Aquatics), were used in this study; 33 fish in experiment 1 and 66 fish in experiment 2. When not actively involved in an experiment, fish were housed alongside stock conspecifics in groups of 15−25 fish in 90 l (40 cm width × 70 cm length × 34 cm depth) glass tanks with a flow-through filtration system. Each tank contained gravel substrate, real and plastic plants and plastic tubes for shelter and enrichment [39]. Tank temperatures were maintained between 26.5°C and 27°C and illuminated with LED bulbs on a 11 : 13 h light : dark cycle. Fish were fed ZM Small Granular pellets (© Copyright 2023 ZM Fish Food & Equipment) 6 days a week.

### 2.2. Ethical statement

All experimental procedures in this study were carried out at the University of Bristol and were approved by the University of Bristol Animal Welfare and Ethical Review Body (UIN/21/003).

### 2.3. Behavioural assays

#### 2.3.1. Experiment 1

Dynamic colour change in zebrafish was investigated by measuring the whole-body luminance of fish following exposure to a white or black environment (a circular arena of 65 cm diameter, approx. 10 cm water depth), where the white treatment had white walls and floor, and the black treatment had black walls and floor. The two treatments represented drastically different visual environments at either end of the luminance spectrum. Similar black and white backgrounds have been used to test for dynamic colour change through changes in luminance in rockpool gobies [14,15]. Thirty-three zebrafish were tested in experiment 1. The testing of an individual fish constituted one trial, and blocks of 10 or 13 consecutive trials constituted one session. For the duration of experiment 1, zebrafish were housed across three tanks (A, B and C) alongside stock conspecifics in groups of around 15 fish.

To begin a session, one of the three housing tanks was randomly selected using a random number generator. A group of 10 (from tanks A and C) or 13 (from tank B) zebrafish were then haphazardly caught from the designated housing tank and moved into a transparent holding tank (14.5 cm width × 33 cm length × 13.5 cm depth), containing an airstone and a plastic plant, adjacent to the experimental arenas. The session would then proceed by conducting 10 or 13 sequential trials, such that every fish in the group was tested. Each fish was tested in one of the two treatments only. The order of treatments was randomly determined under the constraint that 16 fish in total were tested in the white treatment and 17 fish in total were tested in the black treatment. To begin a trial, one fish was haphazardly caught from the holding tank and moved into the designated experimental arena. The fish was allowed to swim freely around the arena for 10 min, constituting the behavioural assay. This length of time was deemed appropriate to acclimate the fish to the arena and induce a colour change following pilot experiments. A white opaque curtain was drawn to separate the experimental arenas from the experimenter for the 10 min duration of the behavioural assay, to avoid disturbance. Once the 10 min had elapsed, the fish was removed from the experimental arena using two small nets and immediately imaged as described in §2.4. The technique for removing the fish from the arena was practised extensively during pilot experiments, such that the process in the present study took around 20–30 s per fish and minimized the time that each fish spent out of water. The imaging process also took around 20 s per fish; therefore, images were taken within a minute from the end of the behavioural assay. No significant colour change was observed in the zebrafish during the imaging process; however, most individuals were observed to revert back to a typical colouration in around 3–5 min after being imaged. Following imaging, the tested fish was placed in a second holding tank identical to the first.

When the entire group of fish had been tested (that is, 10 or 13 individual trials had occurred), the session concluded and all fish were returned to their housing tank. Experiment 1 comprised a total of three sessions over three consecutive days in May 2022.

#### 2.3.2. Experiment 2

In experiment 2, additional predatory and social visual stimuli were introduced to the experimental setup outlined in experiment 1. Fish were tested in the same experimental arenas with a black or white background as previously described. Stimuli were contained in a transparent cylindrical container (17 cm diameter × approx. 20 cm water depth) in the centre of each experimental arena. An individual of the predatory fish species *Channa pleuropthalma* was used as a predatory stimulus. This species was chosen as a predatory stimulus following pilot experiments, in which individuals performed predatory behaviours such as visually tracking the prey. Performance of these behaviours is thought to facilitate recognition of *C. pleuropthalma* as a predatory threat by the zebrafish. Additionally, anti-predator behaviours, including an increase in thigmotaxis in the presence of the predator, were performed by the zebrafish during pilot experimentation. A group of six unfamiliar conspecifics was used as a social stimulus. An identical transparent cylindrical container filled with water was used as a control stimulus. The stimulus containers were non-permeable such that water could not mix between the experimental arenas and stimulus containers, thus excluding odour cues. Combinations of each arena colour and stimulus gave a total of six treatments: White Control, White Predatory, White Social, Black Control, Black Predatory and Black Social.

Sixty-six zebrafish were tested in experiment 2. The testing of an individual fish constituted one trial, and blocks of six consecutive trials constituted one session. For the duration of experiment 2, zebrafish were housed across three tanks (A, B and C) alongside stock conspecifics in groups of approximately 25 fish. The testing order of the three housing tanks was determined by a random number generator prior to any experimentation. Groups of fish from the same housing tank were used in consecutive sessions, and tested fish were returned to the same housing tank following a session. To allow for discrimination between tested and untested fish within the same tank, thus avoiding pseudoreplication, tested fish were contained in fry nets within their housing tank. Fish were released from the fry nets when fewer than six untested fish remained in that housing tank, indicating that no further sessions using fish from that housing tank could be conducted. The group of six conspecifics used as the social stimulus was taken from one of the two housing tanks that the test subject fish was not housed in, as determined by a random number generator (i.e. if the test subject was housed in tank A, the social stimulus could be from tank B or C). This was done to ensure the stimulus fish were unfamiliar to the test subjects, accounting for any effects of familiarity on colour change and behaviour. The *C. pleuropthalma* used as the predatory stimulus was housed in a group of 11 for the duration of experiment 2. It is possible that the same predatory and social stimulus fish were used across multiple sessions, as the identities of the stimulus fish could not be recorded. It is also possible that some zebrafish were both tested as focal fish and used as social stimulus fish at different points in the experiment.

To begin a session, one *C. pleuropthalma* (to be used as the predatory stimulus) was haphazardly caught and placed in one stimulus container, which was filled with water from its housing tank. Six zebrafish (to be used as the social stimulus) were haphazardly caught and placed in a second identical stimulus container, which was filled with water from their housing tank. The third identical stimulus container, used as a control stimulus, was filled with tap water. Six zebrafish (the test subjects) were haphazardly caught from the designated housing tank and moved into a holding tank adjacent to the experimental arenas. Holding tanks were the same as those used in experiment 1, again containing an airstone and a plastic plant. The session then proceeded by conducting six sequential trials, such that every fish in the group was tested. Each fish was tested in one treatment only. The order of treatments was randomly assigned under the constraint that 11 fish were tested in each of the six treatments. When the predatory and/or social stimuli were not in use for any given trial, an airstone at a low bubbling rate was placed inside the containers to ensure the water was kept oxygenated but with minimal disturbance to the fish inside. Furthermore, the water inside each stimulus container was changed frequently throughout the session using water from the fish’s respective housing tank.

To begin a trial, the experimenter would place the designated stimulus container in the centre of the corresponding arena. An opaque cylindrical plastic cover was then placed around the stimulus container, to obscure the stimulus from view of the test subject. A white cover was used in the white arena, and a black cover in the black arena. One zebrafish was then haphazardly caught from the holding tank and moved into the designated experimental arena. The fish were given 3 min to acclimate to the arena. When this time had elapsed, the cover was removed from around the stimulus container. The fish was then given 7 min to swim freely around the arena. The 3 min acclimation time and 7 min free swimming time constituted the behavioural assay, after which the fish was removed from the arena, imaged and placed in a second holding tank as in experiment 1. A white opaque curtain was drawn to separate the experimental arenas from the experimenter for the duration of the behavioural assay, excluding the time in which the experimenter was removing the stimulus cover.

When the entire group of fish had been tested (that is, six trials had occurred), the session concluded. Stimulus fish were released back into their respective housing tanks, and test subjects were placed in fry nets within their housing tanks or released from the fry nets as appropriate. Experiment 2 comprised 11 total sessions across August–September 2022, with maximum two sessions occurring in any one day.

### 2.4. Imaging

Spectrophotometry is often considered a ‘gold standard’ methodology for characterizing animal colouration. Taking accurate spectrophotometer readings of live fish typically requires fish to be handled or restrained *in situ*, or anaesthetized or euthanized and removed from the water. Prolonged handling stress and use of anaesthesia have known effects on fish colouration [14,40,41] and so should be avoided where colouration is being measured, making spectrophotometry impractical for characterizing the colouration of live fish. In the present study, characterization of zebrafish colouration was achieved through image analysis as described in detail in §2.5. Image analysis in this manner represents an alternative methodology to spectrophotometry for measuring fish colouration [42], and requires live fish to be photographed in a standardized manner. Often to produce such standardized images, individual fish are euthanized or dosed with anaesthetic before being removed from the water, laid on a bench or other surface and photographed [30,41,43]. Use of anaesthesia was not feasible in the present study, given the aforementioned effects of anaesthesia on fish colouration. Without delivery of anaesthetic, removing fish from the water for prolonged periods to photograph would cause unnecessary stress to the fish. Euthanising the fish was also not feasible in the present study as fish were retained following experimentation for use in further behavioural experiments. Dynamic colour change has successfully been quantified using image analysis, without the need to deliver anaesthetic, in guppies (*Poecilia reticulata*), rockpool gobies (*Gobius paganellus*) and scorpionfish (*Scorpaena* sp.), by taking continuous video recordings [44] or images at regular intervals [14,16] of the fishes *in situ* with overhead cameras. In the present study, however, colour change as seen from the dorsal view is of less biological interest than that from the lateral view; the characteristic striped zebrafish pattern is most obvious across the lateral surface, and the lateral side would be viewed by both conspecifics and the species’ main aquatic predators. As such, images needed to be taken from the side, rather than overhead, which presents a difficulty in controlling for the distance between the camera and the subject and the orientation of the subject.

To overcome the challenges of photographing live fish in a standardized lateral presentation without the use of anaesthesia, a ‘fish photo booth’ was constructed. The photo booth (25 mm width × 76 mm length × 50 mm height) was constructed from 2 mm thick ultraviolet (UV)-transmitting transparent acrylic glass and sealed with aquarium-safe silicone to ensure it was watertight. It had a sliding inner wall which could be pushed forwards or backwards as desired. In the imaging process, the photo booth would first be filled with water and a fish would be transferred into the photo booth using a small net. The photo booth was then gently placed on a bench immediately adjacent to the experimental arenas, where the camera was set up. The sliding inner wall would be pushed forwards such that the fish was gently restrained between the front and inner wall in a lateral orientation. The fish was held like this for approximately 20 s while several photographs (around 3–4 per fish) were taken. Photographs were taken in RAW using a Canon 550D DSLR camera with a fixed aperture of f/6.3 and an ISO which varied between 100 and 125. Variation in ISO was not systematically associated with any of the treatments. The booth was illuminated with a diffuse broad-spectrum Exo Terra Sunray Metal Halide Bulb (Exo Terra^®^), which emits a broad-spectrum wavelength similar to sunlight. A Kodak grey colour standard at 18% reflectance was attached to the sliding inner wall of the photo booth such that it was visible in every image, underwater and directly behind the fish. This allowed reflectance values of the images to be standardized during image processing. Fish were removed from the booth following imaging by gently submerging the booth into the second holding tank and allowing the fish to swim out. This process of imaging the fish was used in both experiments 1 and 2, immediately following the behavioural assays.

### 2.5. Image analysis

#### 2.5.1. Experiment 1

During the experiment, one individual in the black treatment was visually identified as a leopard zebrafish morph: a phenotypic mutant with spotted rather than striped skin [18,45]. This individual was excluded from the analysis as its patterning could not be compared with that of its striped counterparts. Thus, 32 fish were included in the analysis; 16 in each of the black and white treatments.

**Figure 1.**
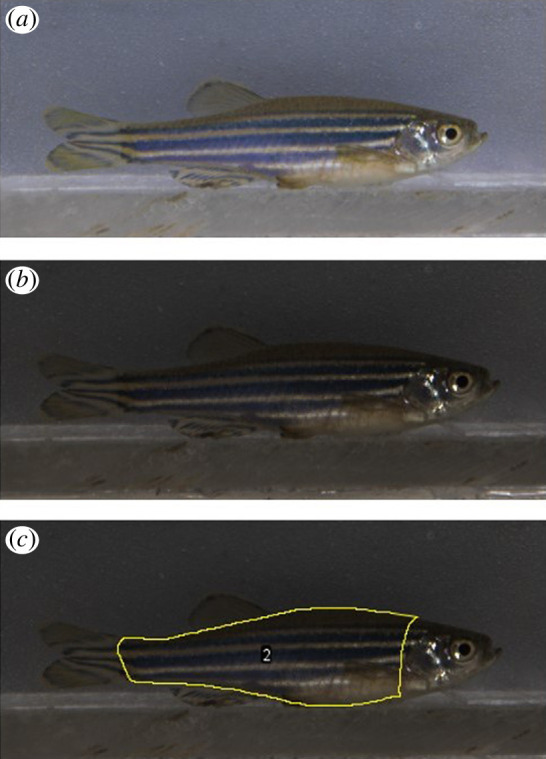
Image processing and ROI selection in micaToolbox for ImageJ. RAW images (*a*) are converted into standardized mspec images and viewed in the micaToolbox interface as linear normalized reflectance stacks (*b*). The ROI is drawn around the fish’s trunk, excluding the head, operculum and fins other than the pectoral fin (*c*). The ROI can then be isolated for image analysis.

Image analysis was conducted using the Multispectral Image Calibration and Analysis Toolbox (micaToolbox) v2.2.1 [46] in ImageJ v1.54d [47]. One RAW image was selected for each fish based on clarity of the subject. RAW images were loaded into the plugin and converted into calibrated multispectral (mspec) images using the Kodak grey standard as a reference of known luminance (18% reflectance) in each image (figure 1*a,b*). A digital scale bar was added to each image, using the same Kodak grey standard as a reference point of known length. The body of each fish was outlined and isolated as a region of interest (ROI; figure 1*c*), as per the ‘worked examples’ supplementary material of van den Berg *et al*. [48]. The fish’s head and operculum were excluded from the ROI, as the dark pupil of the eye and any reflective bright spots caused by opercular movement could affect the mean luminance values. Furthermore, caudal fins, dorsal fins and anal fins were not included in the ROI since these are largely transparent (and thus risk creating luminance artefacts due to the standardized grey background), and varied in the degree to which these fins were clamped or fanned out across different individuals, making standardized comparisons difficult.

Images were not converted to any specific cone-catch model (which can be used to measure colour relative to a given species’ visual system [49]), as there is an overwhelming lack of literature describing the spectral sensitivities of any relevant predatory species; thus cone-catch models could not be generated for any such species. Zebrafish visual systems are better understood [50], and it is known that zebrafish have tetrachromatic colour vision and are sensitive to UV light [51]. However, images were not taken using UV-sensitive camera equipment and so could not be modelled with a zebrafish visual system. In the absence of a complete spectral sensitivity model for the relevant receivers, we conducted our analyses using measures of luminance. Luminance perception is entirely achromatic, and is therefore robust to species differences in colour vision and UV sensitivity [52]. Changes in luminance are relevant to the visual ecology of zebrafish and their predators, given that zebrafish colouration is largely greyscale with broad spectral reflectance, and is perceived primarily through the luminance channel. In the calibrated mspec images, high luminance values represent bright colours and low luminance values represent dark colours, relative to the grey standard. In fishes, the luminance channel is thought to receive input from either the long-wave cone or a combination of the long- and medium-wave cones [52,53]. Therefore, the luminance analysis was conducted through the single long-wave red (visible:R:normalized) channel. Mean luminance values for each fish’s ROI were measured using the Batch Multispectral Image Analysis function of micaToolbox, with the following parameters specified: bandpass filter method = difference of Gaussians (DoG), start size = 2, end size = 512, step size = 1.41421356, step multiplier = mulitply, luminance bands = 0 (off), lowest luminance = 0, highest luminance = 100, transform luminance = linear. To avoid creating false data through image up-scaling, the analysis for experiment 1 was conducted with a scale of 32 px mm^−1^ as determined by the minimum scale for the batch of images.

#### 2.5.2. Experiment 2

Analysis of mean luminance for experiment 2 was conducted in an identical manner as described for experiment 1. The only parameter that differed between the two experiments was scale, where the analysis for experiment 2 was conducted with a scale of 33 px mm^−1^ as determined by the minimum scale for the batch of images. One image from the Black Social treatment was highlighted as a scale outlier and excluded from the analysis, leaving 65 total images in the analysis: 10 in the Black Social treatment and 11 in each of the other treatments.

Additional luminance metrics were generated for each fish’s ROI by plotting a histogram of the grey values in the ROI, using the ‘Histogram’ function in imageJ. Grey values represent pixel intensities in the greyscale colourspace, which are calculated by combining the RGB values of each pixel to give a single intensity value. Each fish’s ROI produced a histogram with two peaks. The grey values at the maxima of each peak were recorded such that the higher value *L*_*max*_ represented the mean luminance of the light areas of the ROI (corresponding to the fish’s light stripes), and the lower value *L_min_* represented the mean luminance of the dark areas of the ROI (corresponding to the fish’s dark stripes). The luminance contrast (i.e. the relative difference between the mean luminance of the dark and light stripes), specified as Weber contrast *C_W_*, was then calculated for each fish with the following equation:


$$C_{W}=\frac{L_{max}-L_{min}}{L_{min}}.$$

### 2.6. Statistical analysis

All analyses were conducted using R v4.2.1 [54] in RStudio v2022.07.2 [55].

#### 2.6.1. Experiment 1

To test for any effect of arena colour on mean luminance, a generalized linear model (GLM) with Gaussian error distribution was fitted with mean luminance as the response variable and arena colour as the predictor variable. To account for any effect of testing order (i.e. session number) on mean luminance, two additional candidate GLMs were fitted either with session as a single predictor, or with arena colour as a main effect and session as a fixed effect. Finally, a null model with no explanatory variables was fitted. The model assumptions of normality of residuals and homogeneity of variance were validated for each candidate GLM by visually assessing Q–Q and residual versus fitted plots, respectively.

Aikake information criterion corrected (AICc) for small samples was used to compare model fits of the candidate GLMs using the ‘aictab’ function in the R package ‘AICcmodavg’ [56]. AICc model selection compares the goodness-of-fit of specified models, assigning AICc values such that *∆*AICc = 0 indicates the most likely model given the data. Support is also given to any models with AICc values within two AICc units of the most likely model and more than two AICc units less than the null model. Of the supported models, simpler models are favoured as the most likely models given the data. The explanatory variables within any supported models may be considered important in predicting the response variable.

#### 2.6.2. Experiment 2

Analyses were conducted to test for any effects of arena colour, stimulus and session on the luminance metrics mean luminance, light stripe luminance, dark stripe luminance and luminance contrast. For each of the response variables, several candidate GLMs with Gaussian error distribution were fitted: a global model containing the main effects of arena colour and stimulus, session as a fixed effect and an arena colour × stimulus interaction term; a model containing all main and fixed effects; a model containing only the main effects of arena colour and stimulus; models with arena colour, stimulus and session as single predictors; and a null model with no explanatory variables. Assumptions of normality of residuals and homogeneity of variance were validated for each model by visually assessing QQ and residual versus fitted plots.

For each response variable, the corresponding candidate GLMs were compared with AICc model selection as detailed above. Where the most likely model given the data, as determined by AICc model selection, contained a categorical response variable with more than two levels, *post hoc* pairwise comparison of estimated marginal means was conducted using the R package ‘emmeans’ [57].

A Pearson’s correlation test was conducted to test for a correlation between dark stripe luminance and light stripe luminance. The two variables were both normally distributed.

## 3. Results

### 3.1. Experiment 1

**Figure 2.**
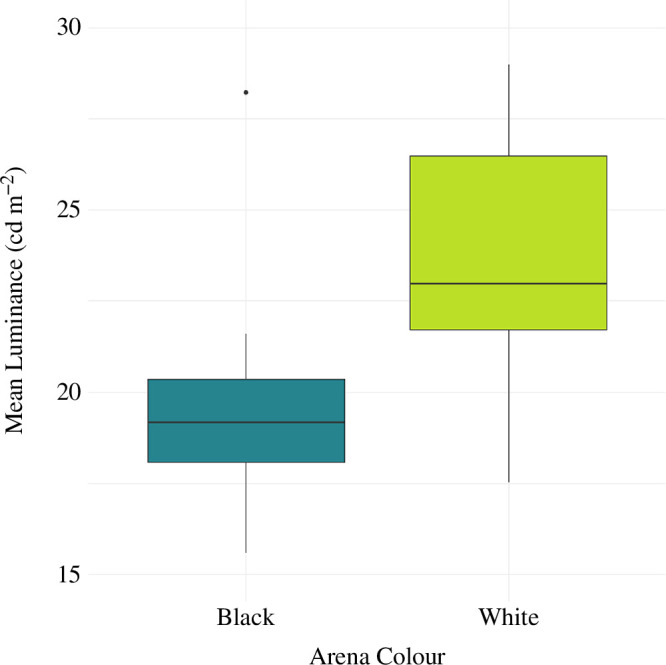
Mean luminance of zebrafish immediately following the behavioural assay, as a function of the colour of the experimental arena. Fish in the white treatment (*n* = 16) had a higher mean luminance than fish in the black treatment (*n* = 16). Boxes are plotted from the first to the third quartiles, with the median indicated by the thicker horizontal line. Whiskers extend to 1.5 times the interquartile range beyond the box edges. Values outside 1.5 times the interquartile range are considered outliers, and are represented by dots.

**Figure 3.**
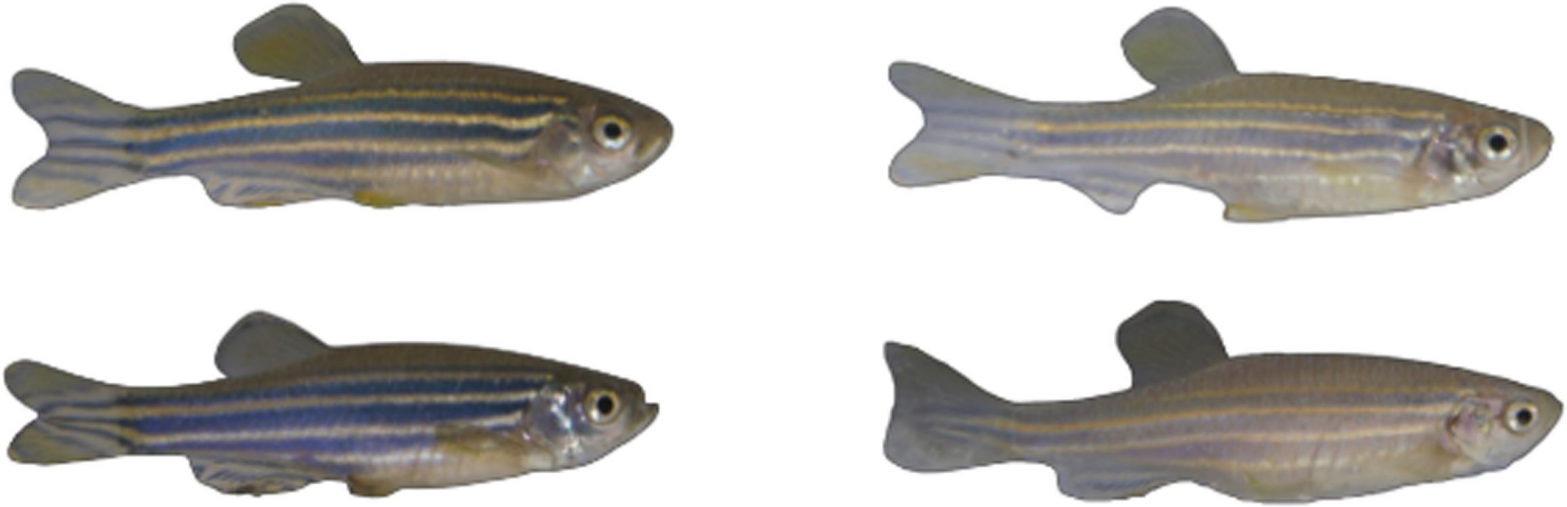
Example images of four individual zebrafish displaying a typical colouration immediately following the behavioural assay in the black (left two images) and white (right two images) treatments. All images were taken under identical lighting conditions.

AICc model selection indicated strong support for arena colour as a predictor of mean luminance; the model with the main effect of arena colour was the most likely model given the data, with an AICc value more than two units lower than that of the null (table 1). There was no support for an effect of session order on mean luminance. In this case, fish in the white treatments had, on average, a higher mean luminance than fish in the black treatments (figures 2 and 3).

**Table 1.** AICc model selection for GLMs with the specified explanatory variables and mean luminance as the response variable, for experiment 1. Models are listed by likelihood given the data, where *∆*AICc represents the difference in AICc values between models, and *∆*AICc = 0 indicates the most likely model. d.f. indicates the degrees of freedom.

explanatory variables	*∆*AICc	d.f.
arena colour	0.00	3
arena colour + session	4.34	5
1 (null model)	9.65	2
session	11.8	4

### 3.2. Experiment 2

**Figure 4.**
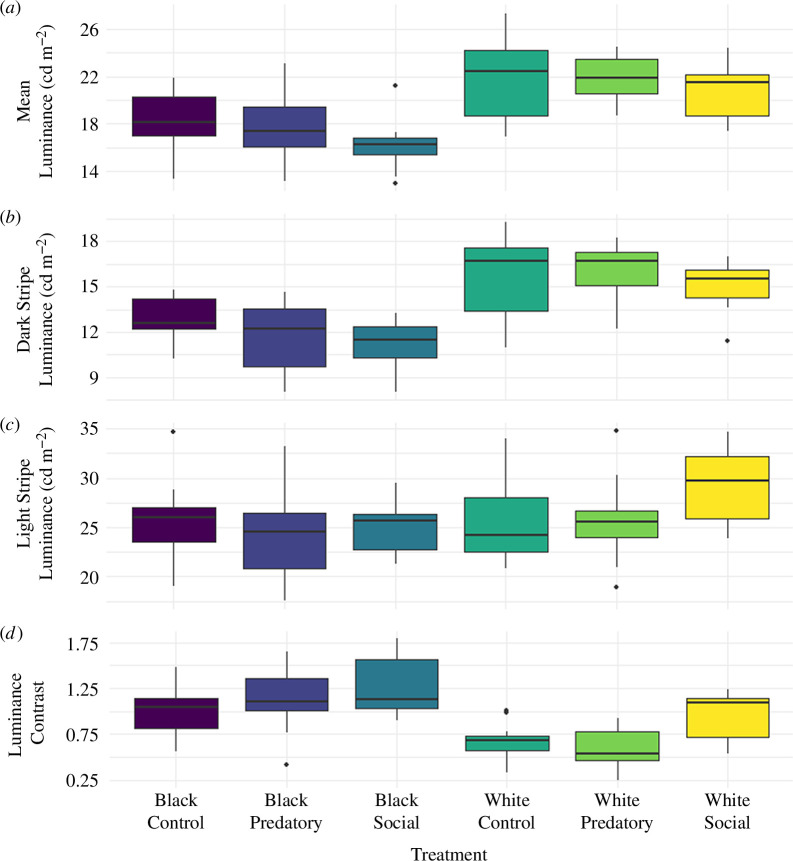
Measures of zebrafish luminance immediately following the behavioural assay, plotted per treatment. Boxes are plotted from the first to the third quartiles, with the median indicated by the thicker horizontal line. Whiskers extend to 1.5 times the interquartile range beyond the box edges. Values outside 1.5 times the interquartile range are considered outliers and are represented by dots. (*a*) Mean luminance per treatment. Fish in the white treatments (White Control *n* = 11; White Predatory *n* = 11; White Social *n* = 11) had a higher mean luminance than fish in the black treatments (Black Control *n* = 11; Black Predatory *n* = 11; Black Social *n* = 10). There is no effect of stimulus on mean luminance. (*b*) Dark stripe luminance per treatment. Fish in the white treatments had a higher mean luminance than fish in the black treatments. There is no effect of stimulus on dark stripe luminance. (*c*) Light stripe luminance per treatment. There is no effect of arena colour or stimulus on light stripe luminance. (*d*) Luminance contrast per treatment. Fish in the black treatments had a higher luminance contrast than fish in the white treatments. Fish in the social treatments had a higher luminance contrast than fish in the control or predatory treatments.

To predict mean luminance, AICc model selection indicated strong support for the model containing arena colour as a single predictor (table 2). Support was also given to the model containing the main effects of arena colour and stimulus, as this was within two AICc units of the most likely model (table 2). However, the simpler model is favoured by AICc as the most likely model given the data. Furthermore, the model containing stimulus as a single predictor was not supported by AICc model selection (table 2), suggesting that the support for the model containing the main effects of arena colour and stimulus is likely explained by the predictive power of arena colour alone. Arena colour is therefore determined to be the best predictor of mean luminance, where fish in the white treatments had a higher mean luminance than those in the black treatments (figure 4*a*). There was no support for an effect of the session on mean luminance.

**Table 2.** AICc model selection for GLMs with the specified explanatory variables and mean luminance as the response variable, for experiment 2. Models are listed by likelihood given the data, where *∆*AICc represents the difference in AICc values between models, and *∆*AICc = 0 indicates the most likely model. d.f. indicates the degrees of freedom.

explanatory variables	*∆*AICc	d.f.
arena colour	0.00	3
arena colour + stimulus	0.95	5
arena colour + stimulus + session	23.07	15
1 (null model)	28.59	2
arena colour + stimulus + session + arena colour × stimulus	29.53	17
stimulus	31.13	4
session	50.35	12

The main effect of arena colour was determined to be the best predictor of dark stripe luminance according to AICc model selection (table 3). Support was also given to the model containing the main effects of arena colour and stimulus (table 3); however, the simpler model containing arena colour as a single predictor is again favoured as the most likely model given the data. No support was given for an effect of session on dark stripe luminance. Here, the mean luminance of a fish’s dark stripes was higher for fish in the white treatments than fish in the black treatments (figure 4*b*).

**Table 3.** AICc model selection for GLMs with the specified explanatory variables and dark stripe luminance as the response variable. Models are listed by likelihood given the data, where *∆*AICc represents the difference in AICc values between models, and *∆*AICc = 0 indicates the most likely model. d.f. indicates the degrees of freedom.

explanatory variables	*∆*AICc	d.f.
arena colour	0.00	3
arena colour + stimulus	1.64	5
arena colour + stimulus + session	26.17	15
arena colour + stimulus + session + arena colour × stimulus	31.18	17
1 (null model)	37.41	2
stimulus	40.55	4
session	60.93	4

Of the candidate GLMs with the response variable light stripe luminance, AICc model selection also identified the model containing arena colour only as the most likely model given the data (table 4). However, the most likely model was within two AICc units of the null model. As such, none of the explanatory variables are considered to have strong support in predicting the luminance of the light stripes (figure 4*c*).

**Table 4.** AICc model selection for GLMs with the specified explanatory variables and light stripe luminance as the response variable. Models are listed by likelihood given the data, where *∆*AICc represents the difference in AICc values between models, and *∆*AICc = 0 indicates the most likely model. d.f. indicates the degrees of freedom.

explanatory variables	*∆*AICc	d.f.
arena colour	0.00	3
1 (null model)	1.11	2
arena colour + stimulus	1.22	5
stimulus	2.22	4
session	19.89	12
arena colour + stimulus + session	22.11	15
arena colour + stimulus + session + arena colour × stimulus	26.61	17

A Pearson’s correlation test indicated a positive linear correlation between dark stripe luminance and light stripe luminance (*r* (63) = 0.53, *p* <0.001), which was conserved across arena colour and stimulus groups.

The best predictors of luminance contrast between the light and dark stripes given the data were the main effects of arena colour and stimulus, with no additional support for an effect of session, according to AICc model selection (table 5). In this case, fish had a higher luminance contrast in black treatments compared with white treatments (figure 4*d*). Furthermore, fish in social treatments had significantly higher luminance contrast than in predatory treatments (*t* (61) = −2.813, *p* < 0.05) or control treatments (*t* (61) = −3.156, *p* < 0.01), with no significant difference in luminance contrast between fish in predatory and control treatments (*t* (61) = −0.346, *p* > 0.05 ; figure 4*d*).

**Table 5.** AICc model selection for GLMs with the specified explanatory variables and luminance contrast as the response variable. Models are listed by likelihood given the data, where *∆*AICc represents the difference in AICc values between models, and *∆*AICc = 0 indicates the most likely model. d.f. indicates the degrees of freedom.

explanatory variables	*∆*AICc	d.f.
arena colour + stimulus	0.00	5
arena colour	6.96	3
arena colour + stimulus + session	22.21	15
arena colour + stimulus	25.58	4
arena colour + stimulus + session + arena colour × stimulus	27.49	17
1 (null model)	28.35	2
session	50.74	12

## 4. Discussion

Despite a thorough understanding of the development and regulation of zebrafish colouration, few studies on this species have yet characterized rapid dynamic colour change as a behavioural response to the visual environment. In the present study, we observed dynamic colour change in zebrafish, quantified as a change in within-body and whole-body luminance, over the course of a 10 minute behavioural assay. In fishes, physiological colour change that occurs over short time frames (from milliseconds to a few minutes) is controlled via neurally regulated rapid intracellular translocation of pigment granules within chromatophore cells [2]. There is also some indication that the rapid physiological chromatophore response is hormonally regulated in zebrafish, where intra-melanophore motility responds to MCH and MSH [4]. While we did not investigate the mechanisms of dynamic colour change here, it is highly likely that physiological colour change was induced in this instance, given the rapid changes in luminance that were observed.

We demonstrated context-dependent dynamic colour change in zebrafish as consistent with previous reports [4,21]. This suggests that use of a ‘fish photo booth’ for imaging live fish can produce reliable, standardized images of live fish, suitable for quantifying dynamic colour change with subsequent image analysis. Importantly, the methodology used here eliminates the requirement for anaesthetic in the imaging process, allowing for an accurate representation of fish colouration at the point immediately following a behavioural trial. The methodology also aimed to minimize handling time of the test subjects; however, it did still require fish to be netted and transported between the experimental arena and the photo booth prior to imaging.

Changes in zebrafish mean luminance were best predicted by the background colour of the environment (i.e. arena colour), regardless of additional social or predatory stimuli. Changes in mean luminance appear to be driven by changes in dark stripe luminance, which were predicted by arena colour. No luminance changes in response to the visual environment were observed in the zebrafish’s light stripes. As melanophores are present in zebrafish dark stripes and absent in zebrafish light stripes, the melanophore response likely plays an important role in mediating zebrafish dynamic colour change, as is consistent with current knowledge of dynamic colour change and pigment regulation in this species [4,21].

Changes in mean luminance occurred in the expected direction relative to the colour of the visual environment: fish lightened or darkened in response to a light or dark background, respectively. These findings are congruent with crypsis through background matching as a hypothesized function of dynamic colour change in zebrafish. Background matching is a form of protective colouration which functions as an anti-predation strategy to decrease an individual’s risk of detection (and subsequent attack) by predators [58]. Background matching is often advantageous where a species’ natural environment is visually stable. Zebrafish, however, occupy visually heterogeneous environments, with a high degree of variation in vegetation cover, water depth and water quality [20]. Where the visual environment is variable, the ability to match numerous backgrounds by rapidly changing colour provides an obvious adaptive advantage [59]. For instance, light colouration and luminance may facilitate background matching of a zebrafish against the well-lit banks of a shallow pond; however, this colouration could render the same individual highly conspicuous to predators as it moves into a deeper, more densely planted area of the same habitat. Transient background matching as a function of dynamic colour change in zebrafish is therefore consistent with the ecology of the species, allowing crypsis to be maintained as an individual traverses its entire environment. Although background matching is a likely strategy for zebrafish to reduce detection risk, we cannot conclude that zebrafish can effectively achieve crypsis through background matching in their natural environment without evaluating how closely their mean luminance (or overall colouration) matches that of their natural backgrounds when viewed through the visual systems of their predators.

Presence of a predatory stimulus was not identified as a predictor of zebrafish mean luminance, contrary to expectations. This could indicate that the predatory stimulus was not perceived as an immediate predatory threat. However, even aquarium-reared domestic zebrafish possess an innate predator recognition ability, and will perform anti-predator behaviours in the presence of sympatric predatory species, even without prior exposure to such predators [60,61]. Observations taken during pilot experimentation noted that the zebrafish did respond to the visual stimulus of a live *C. pleuropthalma* by performing typical anti-predator behaviours, such as thigmotaxis. It is likely that zebrafish mean luminance was not predicted by the predatory stimulus owing to the colour change response to the background reaching a ‘ceiling’. Here, the luminance changes were maximized in the arenas even in the control treatment without additional stimuli. Such a response could have been due to the arenas being perceived as high-risk environments, regardless of the additional stimuli.

Both arena colour and the visual stimulus of a group of conspecifics were identified as predictors of luminance contrast (i.e. the the relative difference in mean luminance between a zebrafish’s dark and light stripes) in zebrafish. Changes in luminance contrast appear to be driven by subtle luminance changes in both the light and dark stripes; in the social treatments, for example, it appears that zebrafish dark stripes become marginally darker and light stripes become marginally lighter than in the predatory or control treatments. These subtle luminance changes were not statistically significant where light or dark stripe luminance were analysed in isolation; however, the differences were detected in the statistical analysis when the analysis was conducted with a relative measure of luminance contrast between the stripes. As such, the biological importance of taking relative measures (in this case the relative luminance difference between light and dark stripes) alongside absolute measures (in this case light and dark stripe luminance alone) is highlighted, so as to not overlook any biologically relevant effects. Zebrafish light stripes are populated by xanthophores and iridophores only [62], thus luminance changes here must be occurring independently of the melanophore response. Although less well understood, it is known that both xanthophores and iridophores possess cellular motility of pigment granules and guanine platelets, respectively [12,63–67]. As such, we suggest that the response of one or both of these cell types plays some role in mediating dynamic colour change in zebrafish, in addition to the known melanophore response.

Changes in luminance contrast were predicted by arena colour, where luminance contrast was greater for fish in black treatments than fish in white treatments. This result likely emerged as a result of physiological constraints on the proposed xanthophore or iridophore response; as the dark stripes become darker in a black visual environment, the light stripes, which lack the melanophores responsible for dark colouration, remain relatively light in comparison, and the luminance contrast between the two stripes increases.

We also identified social context as an important predictor of luminance contrast, where luminance contrast was increased in the presence of conspecifics. Longitudinal contrasting stripes have been hypothesized to play a role in social signalling in some fishes, since this pattern is associated with species demonstrating strong social tendencies [29,68,69], and conspicuous differences in luminance contrast can be reliably detected underwater [70]. Moreover, stripes have been identified as an important cue for group assortment and social behaviour in zebrafish [34,37]. This considered, it is a probable hypothesis that zebrafish stripes function in social signalling. Since luminance contrast increased in the presence of conspecifics, we suggest that dynamic colour change may be used to mediate the quality of these signals through changes in internal contrast.

Although likely, we cannot conclude that zebrafish stripes function exclusively in social signalling; zebrafish stripes could serve an alternative or additional anti-predator function. Contrasting stripes on an animal in motion have been hypothesized to function in ‘dazzle colouration’, which Scott-Samuel *et al.* [71] define as a defensive colouration which ‘interferes with the interception of a moving object, due to perceptual distortions of target speed, trajectory and/or range’. Moreover, Hogan *et al.* [72,73] suggest that dazzle colouration might enhance the anti-predatory benefits of the confusion effect. The confusion effect, where the likelihood of predatory success decreases when attacking moving prey within a large group [74], is often attributed to sensory and cognitive limitations causing a reduction in a predator’s ability to isolate and track an individual prey within a large aggregation [75,76]. We therefore consider that an increased internal contrast of zebrafish stripes in the presence of conspecifics, as was seen in the present study, could potentially mediate dazzle colouration and the confusion effect for shoaling zebrafish, to deter or reduce the accuracy of an attack on an individual within a shoal. Empirical evidence of dazzle colouration in biological systems, particularly where dazzle colouration might augment the effects of the confusion effect, is limited to only a few studies at present [17,71]. Additionally, it is not well understood whether dazzle colouration is most effective for targets with high-contrast or low-contrast patterns, or if the influence of contrast is context-dependent [71,73,77]. Thus, further work must be undertaken to determine whether longitudinal stripes might facilitate the dazzle effect in shoaling fish such as zebrafish, and whether this might be mediated by dynamic colour change.

It is generally understood that the evolution of animal colouration occurs under the influence of numerous selection pressures. A prey animal might therefore be required to navigate a trade-off between maintaining a cryptic colouration versus producing visually conspicuous social signals, due to the increased predation cost of being conspicuous [38,78]. Zebrafish may be able to navigate this trade-off to some extent through transient social signalling, as facilitated by dynamic colour change [79], by increasing their internal contrast when around conspecifics to produce a temporarily more conspicuous social signal. This should allow zebrafish to maximize detection by conspecifics when shoaling, without compromising on the ability to maintain crypsis when alone. Moreover, the numerous anti-predator benefits of being in a group [74] might outweigh the potential increased detection costs of producing conspicuous signals when shoaling [80]. Furthermore, considering the alternative hypothesis that zebrafish stripes may function in dazzle colouration, dynamic colour change could allow zebrafish to use two distinct protective colouration strategies in different social contexts. Background matching is likely to be an effective cryptic colouration strategy for reducing the detection of solitary fish. However, for groups of cryptic prey, detectability typically increases with increasing group size [81,82]. Zebrafish may therefore favour crypsis when alone, and favour dazzle colouration as an alternative defensive colouration strategy when shoaling. We do note that the luminance contrast of zebrafish stripes was overall higher in the black treatments than the white treatments, and so differences in internal contrast in the presence and absence of conspecifics are less pronounced in the black treatments. This could indicate that zebrafish might not be able to navigate the trade-off between cryptic and conspicuous colouration through dynamic colour change as efficiently in darker environmental conditions. However, the predation costs of conspicuous high-contrast stripes may be less relevant in darker habitats, if low light levels render it functionally more difficult for a visual predator to detect a zebrafish [83]. Similarly, zebrafish may rely on behaviour instead of (or in addition to) colour change to navigate the trade-off in these conditions; it is likely that for zebrafish, darker habitats equate to more vegetation-dense habitats, so they are perhaps better protected from predation by using vegetation as physical refuges. Further experimental confirmation and visual modelling are needed to determine the relationship between crypsis, behaviour and the social environment in this and other species [22].

Although it is logical to assume that there is always a trade-off between cryptic and conspicuous colour patterns, it is also entirely possible that these ‘conflicting’ colouration strategies can occur simultaneously. Owing to differences in the visual systems and typical viewing distances between different receivers, colour patterns which are highly conspicuous to conspecifics may be simultaneously perceived as cryptic to a predator, thus may not incur increased predation risk [84]. Little is known of the visual ecology of the predatory fish species that are sympatric with zebrafish [85]. However, at the distances from which ambush predators such as these would be first detecting a zebrafish, it is possible that many predators would be incapable of resolving the individual stripes of a zebrafish’s pattern [44,86] as the high spatial frequency information of the narrow contrasting stripes would be lost [87] (see electronic supplementary material, figure S1). Therefore, changes in the luminance contrast between zebrafish stripes may not be of biological relevance from the perspective of a predator during initial detection. Instead, at these distances, predators may be more likely to perceive the mean luminance of the entire fish, due to distance-dependent perceptual blending of the dark and light stripe luminance values [88–90]. In the present study, mean luminance was predicted by arena colour but not by stimulus. This suggests that zebrafish might be capable of achieving crypsis even in a social context, where the mean luminance of their perceptually blended stripes matches the background despite any changes in internal contrast. In comparison to zebrafish predators, the viewing distances relevant to shoaling conspecifics are much closer: members of a shoal typically maintain distances between their nearest neighbours (nearest neighbour distance) of around 0.6−2 body lengths [91], equating to around 2−7.5 cm in wild zebrafish [19]. At these distances, the contrasting stripes of a zebrafish’s pattern are perceived at lower spatial frequencies; thus, it is more likely that shoaling zebrafish will be able to resolve the stripes of a nearby conspecific (electronic supplementary material, figure S1). It is, therefore, possible that zebrafish are able to remain cryptic to predators at a distance, while simultaneously signalling to neighbouring conspecifics, due to distance-dependent effects [86,89,90]. Similarly, zebrafish stripes could also function in multiple distance-dependent defensive colouration strategies [90]. Despite many zebrafish predators likely being unable to resolve a zebrafish’s stripes at the point of initial detection, the predation sequence can be defined across predictable stages (see [17, pp. 5−8]), where the distance between predator and prey decreases in the later stages of the sequence. A predator’s potential to resolve the striped pattern should therefore increase as the predation sequence progresses (electronic supplementary material, figure S1). As such, zebrafish could be perceived as cryptic at a distance, while the contrasting stripes could function in dazzle colouration as a post-detection defensive colouration strategy to deter or reduce the accuracy of an attack once crypsis is broken [92]. To our knowledge, the relationship between distance-dependent perception of striped patterns and dazzle colouration as a defensive colouration strategy has not yet been experimentally evidenced. There is, however, empirical evidence of distance-dependent defensive colouration from (but not limited to) butterfly and moth larvae [93,94], frogs [94] and artificial moth-like prey [95], demonstrating that the same colouration can be perceived simultaneously as both aposematic and cryptic depending on viewing distance of the intended receivers. Additionally, there is some suggestion that conspicuous blue and yellow striped patterns in reef fishes [88] could function in both crypsis and social signalling due to distance-dependent effects; however, the wider occurrence of simultaneous crypsis and social signalling in nature is not well understood [88,96]. We suggest that zebrafish represent a useful candidate model system for further exploration of distance-dependent colouration strategies, with particular emphasis on the interplay between cryptic colouration, social signalling and motion dazzle. Although, we note a need to first develop our understanding of the visual ecology of the relevant zebrafish predators.

To conclude, we demonstrate that zebrafish are capable of rapid physiological colour change, which can be behaviourally induced and reliably quantified through image analysis. Changes in zebrafish mean luminance were best predicted by the colour of the visual environment, indicating that transient background matching is a probable function of dynamic colour change in this species. Social context is also shown to influence zebrafish colour patterns, where the internal contrast of the stripes is increased in the presence of conspecifics. We suggest that this may function in social signalling to enhance signal quality, or to mediate dazzle colouration as a defensive colouration strategy, although these functions may not be mutually exclusive. It is possible that dynamic colour change may help zebrafish navigate the trade-off between cryptic and conspicuous colouration, by potentially allowing different colouration strategies to be used as favoured across different contexts. Additionally, considering likely differences in the relevant viewing distances of zebrafish and their predators, we speculate that high-contrast zebrafish stripes may function in multiple simultaneous distance-dependent strategies. Our results begin to address a current research gap surrounding the ecological functions of dynamic colour change in zebrafish, a biologically important model species. However, for a more complete understanding of the functions of dynamic colour change in zebrafish and in fishes more generally, we believe further exploration of this research area is warranted.

## Data Availability

Data for experiments 1 and 2 are provided as electronic supplementary material [97].
